# Rapid adaptation (or not) in restored plant populations

**DOI:** 10.1111/eva.12959

**Published:** 2020-04-23

**Authors:** Susan M. Magnoli

**Affiliations:** ^1^ W.K. Kellogg Biological Station and Department of Plant Biology Michigan State University Hickory Corners MI USA

**Keywords:** *Chamaecrista fasciculata*, colonizing populations, ecological restorations, rapid adaptation

## Abstract

Mismatches between the traits of a colonizing population and a novel habitat can generate strong selection, potentially resulting in rapid adaptation. However, for most colonization events, it can be difficult to detect rapid adaptation or distinguish it from nonadaptive evolutionary changes. Here, I take advantage of a replicated prairie restoration experiment to compare recently established plant populations in two closely located restored prairies to each other and to their shared source population to test for rapid adaptation. Using a reciprocal transplant experiment six years after the populations were established, I found that one restored plant population showed evidence of adaptation, outperforming the other restored population when grown at its home site. In contrast, I detected no evidence for adaptation at the other site. These findings demonstrate that while rapid adaptation can occur in colonizing plant populations, it may not be the rule. Better understanding of when adaptation may or may not occur in these contexts may help us use evolution to our advantage, potentially improving establishment of desirable species in restored habitats.

## 1 INTRODUCTION

Range expansions driven by climate change, species invasions, and the restoration of degraded landscapes all result in populations colonizing new areas where they may encounter novel abiotic and biotic conditions (Alexander, Diez, Hart, & Levine, 2016; LaRue, Chambers, & Emery, 2017; Mooney & Cleland, 2001). In each of these scenarios, populations are likely to colonize environments to which they are not well‐adapted. As a result, they may experience strong selection (Colautti & Lau, 2015; Kulpa & Leger, 2013; LaRue et al., 2017) and potentially rapidly adapt to conditions in their new habitats (Kinnison, Unwin, & Quinn, 2008; Rius & Darling, 2014; Sax et al., 2007).

While we expect that strong selection on colonizing populations will result in adaptation and while there are numerous examples of rapid evolution occurring during colonization events, few studies differentiate between evolutionary changes due to adaptation and those caused by stochastic processes such as founder effects. Many examples of rapid evolution during colonization come from studies of invasive species where mean trait values differ between native and invasive populations in common gardens (reviewed in Bossdorf et al., 2005; Colautti, Maron, & Barrett, 2009; Felker‐Quinn, Schweitzer, & Bailey, 2013; van Kleunen, Bossdorf, & Dawson, 2018). Because often little is known about the initial size and origin of these populations, it can be difficult to determine whether evolutionary changes are due to selection or founder effects, although several studies of invasive species over well‐sampled geographic clines provide strong evidence that colonizing populations can adapt to climatic factors (e.g., Colautti & Barrett, 2013; Huey, Gilchrist, Carlson, Berrigan, & Serra, 2000; Urbanski et al., 2012). Studies of range expansions in native species have similarly found evidence of rapid, potentially adaptive, evolution in colonizing populations (Macel et al., 2017), while experimental range expansions show that rapid evolution can accelerate population expansion (Williams, Hufbauer, & Miller, 2019).

Here, I examine rapid adaptation in plant populations in recently established habitat restorations. Restorations present an ideal opportunity for studying rapid adaptation in colonizing populations, because unlike range expansions or invasions, we usually know exactly where founding populations originated from and when they were established. In the case of plants, seeds can be saved to compare established populations to their original source to test for evidence of adaptation (Franks, Avise, Bradshaw, Conner, & Etterson, 2008). A demographic boost due to rapid adaptation might also be particularly important to the success of restorations (LaRue et al., 2017), especially because populations sown during restoration may be maladapted as they often come from nonlocal sources (Gallagher & Wagenius, 2016; Vander Mijnsbrugge, Bischoff, & Smith, 2010) and/or are sown into degraded, anthropogenically disturbed environments (Lau, Magnoli, Zirbel, & Brudvig, 2019; Suding, 2011). To test for evidence of rapid adaptation in recently established plant populations in restorations, I capitalized on an experiment in which two former agricultural fields were restored to prairie using identical seed mixes. Six years after restoration, I used a field reciprocal transplant experiment with populations of *Chamaecrista fasciculata* from these sites and seeds saved from their shared source population to determine whether rapid adaptation had occurred.

## 2 METHODS

### 2.1 Study system

*Chamaecrista fasciculata* Michx. (hereafter *Chamaecrista*) is an annual legume native to eastern North America commonly found in prairies and disturbed sites. It is self‐compatible but predominantly outcrossing (Fenster, 1991a) and is pollinated exclusively by bees (Fenster, 1991b). It appears to have a limited seed bank, with >90% of viable seeds in a seed bank study germinating in the first year (Fenster, 1991a). *Chamaecrista* is often included in prairie restoration seed mixes (Grman, Bassett, Zirbel, & Brudvig, 2015), and its establishment may influence the composition of early successional plant communities (Keller, 2014). In 2010, two former agricultural fields in southwest Michigan, Lux Arbor (42°28′23″N, 85°26′50″W; 13 ha) and Marshall (42°26′37″N, 85°18′34″W; 11 ha), were sown with identical prairie seed mixes (containing 19 grass and forb species, including *Chamaecrista*). The *Chamaecrista* seeds in this mix came from a commercial restoration seed supplier and were a cultivated ecotype from Houston County, MN, USA (Shooting Star Native Seeds, pers. comm.), which is 1–2° higher in latitude than the restoration sites in southwest MI, with slightly lower average rainfall. A portion of the seed mix was saved (hereafter referred to as the “original source”). In 2015 (a maximum of six *Chamaecrista* generations since the original seeds were sown), I collected 5–20 seeds from each of 100 *Chamaecrista* individuals selected at 5‐m intervals along five 100 m transects through the center of each site.

Despite being only 15 km apart, the Lux and Marshall restoration sites differ in both abiotic and biotic factors. The Lux site is less productive and more grass‐dominated than the Marshall site (Figure S1), but *Chamaecrista* biomass is consistently higher at the Lux site (2–12 times greater, depending on year; Figure S2). The sites also differ in underlying abiotic factors (Stahlheber, Watson, Dickson, Disney, & Gross, 2016). Compared to the Marshall site, the Lux site has lower available soil nitrogen (1.3 g N/kg and 2.3 g N/kg at Lux and Marshall, respectively), lower soil phosphorus (23.78 ppm and 54.54 ppm, respectively), and higher percent soil moisture. A previous study of these *Chamaecrista* populations found evidence of genetic differentiation between populations in flowering time (the Lux population flowers earlier than the Marshall population, which flowers earlier than the original source population), root nodule formation, and specific leaf area (the Lux population is more likely to produce root nodules than the Marshall population and has significantly lower SLA than the original population; Magnoli & Lau, 2019), suggesting that these populations have undergone evolutionary changes over the six years since they were established.

### 2.2 Reciprocal transplant experiments

To determine whether *Chamaecrista* populations have rapidly adapted to local site conditions, I conducted a reciprocal transplant experiment, growing Lux, Marshall, and the original source plants at both the Lux and the Marshall sites. I grew seeds collected from each site in 2015, along with seeds from the original population, in the greenhouse for one generation to minimize maternal effects. For the Lux and Marshall populations, I grew one seed from each of 96 of the 100 maternal plants. Each of these was randomly assigned to be a sire or a dam, and each sire was used to pollinate two dams, for a total of 64 full‐sibling families (32 half‐sibling families) per site.

Due to low germination of the original source seeds (only 7 seeds germinated), I did not include family structure when pollinating these plants, but instead used one plant as a pollen donor on a given day, so that every plant was crossed with every other plant several times. There was a risk that these small number individuals did not accurately represent the original source population, if long‐term seed viability was correlated with traits relevant for adaptation or if the small sample size led to founder effects (Franks, Sekor, Davey, & Weis, 2019). In a related study comparing mean trait values of this population and the two restored populations (Magnoli & Lau, 2019), I estimated whether founder effects led to observed trait differences between these populations by bootstrapping trait distributions for each trait in the restored populations by repeatedly drawing seven families at random from each population, to calculate a distribution of population mean trait values controlling for sample size. The original source trait means fell outside the 95% confidence intervals of the distributions, indicating that trait differences were likely not the result of a founder effect in my sample of the source population. However, I cannot rule out bias in which seeds survived storage, meaning that fitness comparisons between the restored populations and the original source population should be interpreted cautiously. While this concern pertains to the comparisons with the original source population, a pattern of local adaptation or partial local adaptation in which the Lux population outperforms the Marshall population at the Lux site (or the Marshall population outperforms Lux at the Marshall site) would provide further support for rapid adaptation at least at the local scale, rather than founder effects or bias in which seeds survive storage.

In May 2016, I germinated seeds produced by the greenhouse‐reared plants and, one week later, transplanted seedlings into three 4 m × 4 m plots (each divided into 16 1 m × 1 m subplots with plants spaced 16 cm apart) at both restoration sites [(2 seedlings/extant population full‐sib family × 64 full‐sib families × 2 extant populations + 64 original source population seedlings) × 3 plots × 2 sites; *N* = 1,920 total seedlings]. I disturbed existing vegetation as little as possible while planting seedlings. I monitored survival over the course of the growing season and collected seeds produced by each plant at the end of the season in September 2016. As *Chamaecrista* is an annual, these fitness measures represent an estimate of lifetime fitness.

Because germination rate is an additional important fitness component that I was not able to measure in the reciprocal transplant described above, I conducted an additional reciprocal transplant with seeds from each population the following year. In November 2017, I sowed seeds from each of the three populations into twelve 1 m × 1 m plots (seeds spaced 10 cm apart) at both the Lux and Marshall sites (3 populations × 2 sites × 375 replicates; *N* = 2,250 seeds). To keep track of individual seeds, I glued them to plastic swizzle sticks (Soodhalter Plastics Inc.) with water‐soluble Elmer's glue and placed the swizzle sticks in the ground so that the seeds were just below the soil surface. This way, the seeds detach from the swizzle stick in the moist soil, but germinate next to the stick for easy identification. In May 2018, I censused each plot for germination success.

### 2.3 Statistical analyses

To test for differences in fitness among plant populations at each site, I used aster models (Geyer, Wagenius, & Shaw, 2007; Shaw, Geyer, Wagenius, Hangelbroek, & Etterson, 2008) in R v.3.5.1 (R Core Team, 2018), which allow for unified analysis of multiple life‐history stages with appropriate statistical distributions. The aster model integrated two life‐history stages: survival and seed production (I did not include germination because the germination data came from a separate transplant experiment), to estimate lifetime seed production (my measure of fitness) for each population. I used a Bernoulli distribution for survival and a Poisson distribution for seed production. I fit models using the reaster() function in the aster package (Geyer, 2018) with plant population, site, and their interaction as fixed effects and plot and subplot as random effects. I tested whether the population × site interaction improved the fit of the model by using likelihood ratio tests to compare models with and without the interaction. Upon finding a significant population × site interaction, I separated the data by site and tested for differences between populations at each site using aster models with only population as a fixed effect and plot and subplot as random effects. If population had a significant effect, I conducted pairwise comparisons between each population. For graphical display, I calculated expected values of lifetime seed production and its standard error from fixed‐effect models.

To examine differences between populations in individual fitness components, I analyzed germination, survival, and seed production of surviving individuals separately using generalized linear mixed models in the lme4 and glmmTMB packages in R (Bates, Maechler, Bolker, & Walker, 2015; Brooks et al., 2017). Models included plant population, site, and their interaction as fixed effects and plot and subplot as random effects (except for the germination model, where there was no subplot). For seed production, I included only plants that survived in the analysis to avoid confounding the two fitness components. I used a binomial family distribution for germination and survival, and a zero‐inflated Poisson distribution for seed production. I validated model fit by inspection of simulated residuals using the DHARMa package (Hartig, 2019). I tested significance using type III sums of squares in the ANOVA function in the *car* package (Fox & Weisberg, 2011) with sum contrasts and calculated estimated marginal means and conducted Tukey's post hoc multiple comparisons tests using the *emmeans* package (Lenth, 2018).

As a way of integrating all three fitness components together, I calculated a rough estimate of population growth rate (*λ*) for each population at each site by multiplying mean fitness component values from the individual fitness component models described above. I calculated standard errors via error propagation, as the square root of the sum of the squared relative errors on each fitness component.

## 3 RESULTS

**FIGURE 1:**
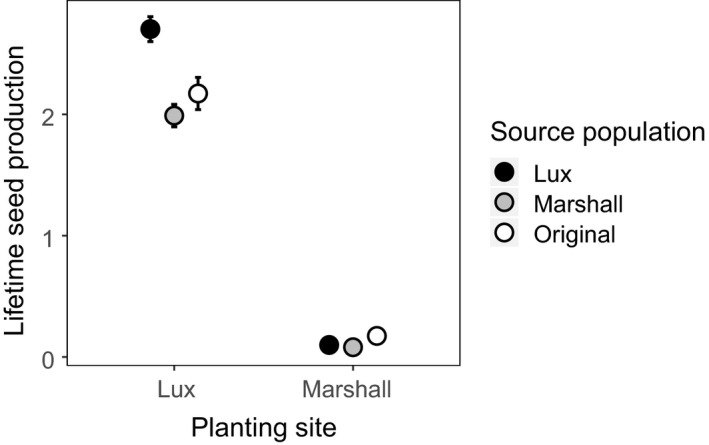
Results from a reciprocal transplant of seedlings in prairie restorations show evidence of rapid adaptation in one population but not another. Points show predicted values (mean ± *SE*) of overall lifetime seed production per plant, based on survival and seed production. Some error bars are obscured by data points. Planting sites are two nearby prairie restorations in southwest Michigan, and source populations are the original population used to seed both sites and the two populations collected from each site 6 years after restoration. Values were predicted using a fixed‐effect aster model, rather than the random‐effect model used to test significance of fixed effects, as parameter estimates from random‐effects models are difficult to interpret

Aster models showed evidence of rapid adaptation in one population but not the other. Populations differed in lifetime seed production, although the magnitude and direction of this effect depended on site (the addition of the population × site interaction term significantly improved model fit; test deviance = 7.15, *p* = .03). Specifically, the Lux population performed better at its home site than the Marshall or original source populations, producing 34% more seeds on average (Figure 1), suggesting rapid adaptation has occurred within 6 years of colonization. In contrast, the Marshall population did not perform best at its home site, where all populations had very low fitness (Figure 1).

**FIGURE 2:**
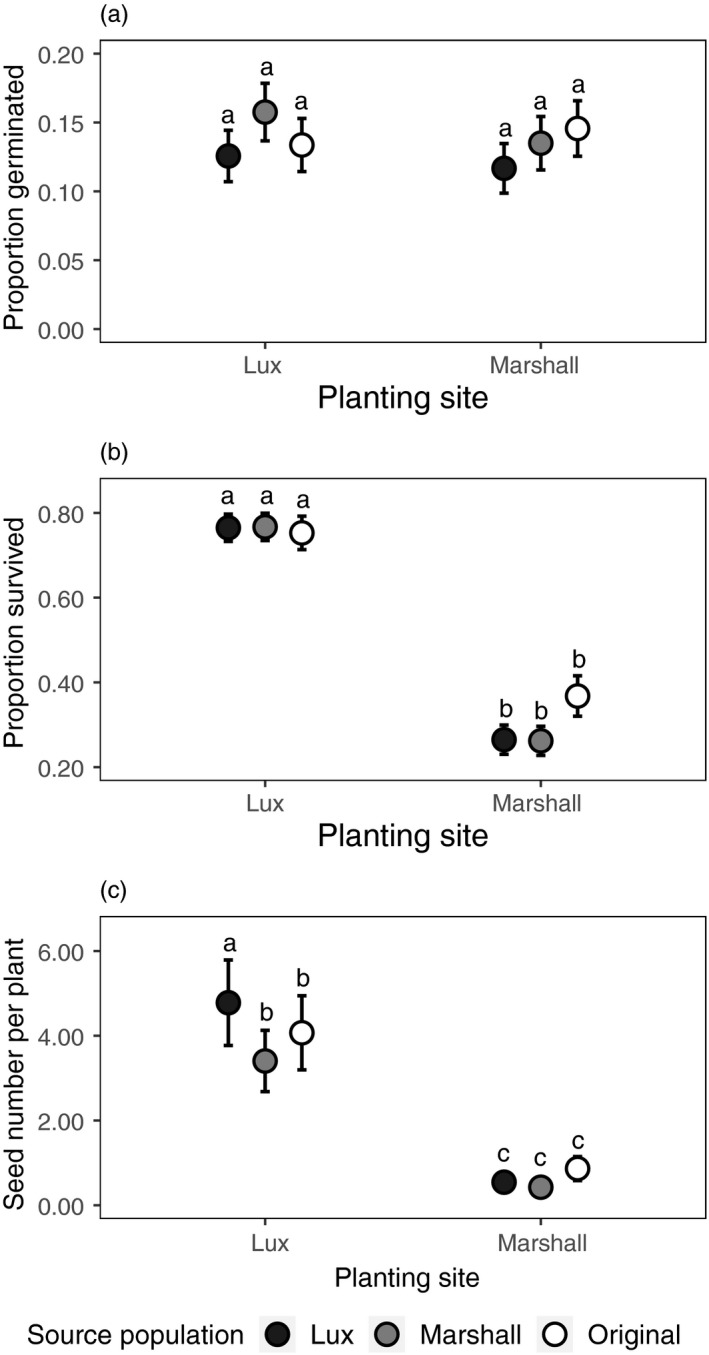
Results from reciprocal transplants of seedlings and seeds in prairies restorations, shown as mean (estimated marginal mean ± *SE*) proportion of seeds that germinated (a), plants that survived to flower (b), and number of seeds produced given the plant survived to flower (c). In (c), some error bars are obscured by data points. Populations differed in seed production but not germination or survival. Models included source population, site, and their interactions as fixed effects and plot as a random effect. Different letters indicate statistically significant differences at *p* < .05

Analyses of individual fitness components showed no significant site x population effects (Tables S1–S3; Figure 2), but the Lux population had higher seed production than the Marshall population, regardless of site (*χ* ^2^ = 8.23, *p* = .02; Table S3). Although the site × population interaction was not significant, the greater seed production of the Lux population was especially notable at the Lux site (Figure 2c), suggesting that the adaptation I detect at Lux is likely driven by increased seed production rather than changes in survival.

Estimates of population growth rates showed that while the Lux population had a slightly higher growth rate than the other populations when grown at the Lux site (Lux site: Lux *λ* = 0.47 ± 0.12, Marshall λ = 0.42 ± 0.11, original λ = 0.40 ± 0.11; Marshall site: Lux *λ* = 0.02 ± 0.007, Marshall *λ* = 0.01 ± 0.004, original *λ* = 0.05 ± 0.019), growth rates appear to not differ dramatically between populations. Difference between populations was minimal because the higher Lux seed production was counteracted by higher Marshall seed germination.

## 4 DISCUSSION

In a study of two recently established plant populations and their shared original source population, I found evidence of rapid adaptation in one population but not the other. Given that both restored plant populations in this experiment originated from the same source population and were planted using the same restoration methods at each site (identical seed mix and seed density, timing of seed sowing, and prerestoration site preparation), this finding begs the question of what factors promote and limit adaptation in colonizing populations.

Adaptation can be limited by several factors. In Antonovics' classic paper (1976), he discussed these factors as constraints to adaptation in marginal populations, but they are likely applicable to colonizing populations as well. Lack of genetic variation can inhibit adaptation (Bradshaw, 1991; Connallon & Hall, 2018), as can small population sizes, which makes populations more susceptible to swamping effects of gene flow and more likely to experience strong genetic drift. Adaptation can also be limited by trade‐offs, if the direction of selection on traits is opposed by the genetic covariance between those traits (Connallon & Hall, 2018; Etterson & Shaw, 2001; Walsh & Blows, 2009). Coevolving species interactions may slow adaptation (coevolutionary constraint), if there are negative correlations between traits mediating interactions with multiple other species (e.g., Wise & Rausher, 2013). Finally, time can be a constraint if populations cannot adapt rapidly enough before going extinct (Bradshaw, 1991; Jump & Peñuelas, 2005; Shaw & Etterson, 2012).

Of the many factors that can constrain adaptation, some are more likely to influence adaptation in my system than others, especially considering the differences between the two restoration sites. First, lack of genetic variation could potentially be a constraining factor. The traits that contribute to adaptation may differ across sites, so while there was clearly enough genetic variation present in the original source population to allow adaptation to occur in the Lux population, if there was a lack of variation in the particular traits that would contribute to adaptation at the Marshall site, this could potentially constrain adaptation. Second, differences between the sites in several biotic factors (including plant community composition [Stahlheber et al., 2016] and rhizobia quality [Magnoli & Lau, 2020]) suggest that coevolutionary interactions between *Chamaecrista* populations and other species could differ between sites. These differences in biotic interactions between sites could potentially lead to coevolutionary constraints that limit adaptation at the Marshall site but not the Lux site.

Population size could also potentially contribute to differences in adaptation between the two restoration sites. The Marshall population has been consistently smaller than the Lux population, based on yearly biomass sampling (Figure S2). If smaller population size resulted in lower genetic diversity (Leimu, Mutikainen, Koricheva, & Fischer, 2006) in the Marshall population, the Marshall population may have been less able to respond to selection than the larger, more genetically diverse Lux population. However, without accurate estimates of population size at these sites, conclusions about the influence of population size on adaptation are limited. It is less likely that trade‐offs limit adaptation in this system. Correlation tests between family mean survival and fecundity for each population grown at its home site show little correlation between these two fitness components (Marshall: *r* = −.04, *p* = .76; Lux *r* = −.006, *p* = .96), suggesting trade‐offs might not explain the lack of adaptation. However, there could be trade‐offs between germination and these traits that I could not assess with this experimental design. Trade‐offs between other traits also could limit adaptation in Marshall but not Lux if selection favors different traits at the two sites or if the expression of genetic covariances differs across sites (environmental effects on the G‐matrix, Wood & Brodie, 2015). While previously observed differences in trait values between the Lux and Marshall populations suggest that selection differed between populations in the past, an earlier study detected little evidence that current selection differs across site (Magnoli & Lau, 2019) or that G‐matrices vary across populations (unpublished data, S.M.M.). Swamping effects of gene flow are also unlikely to limit adaptation, because there are no other known naturally occurring *Chamaecrista* populations in the surrounding landscape.

The scale of the adaptation I found in the Lux population appears to be very local, with this population performing better than its original source population and the Marshall population at its home site but not at the other nearby site. While I found no evidence of local adaptation in this system in the strict sense (as the Marshall population did not outperform the others at its home site), local adaptation studies that focus on the scale of adaptation can provide context for my results. In a review of local adaptation studies in plants, Leimu and Fischer (2008) found that the strength of local adaptation was not tightly associated with geographic distance between populations, suggesting that adaptation over small geographic scales is not uncommon. Further supporting the idea that adaptation can occur at small scales, studies of selection over small geographic distances have found that selection can differ in both strength and direction over very small (<100 m) distances (e.g., Kalisz, 1986). In contrast, in a reciprocal transplant study of naturally occurring *Chamaecrista fasciculata* populations, Galloway and Fenster (2000) found local adaptation only at large spatial scales (populations >1,000 km apart) and suggest that local adaptation may be limited by metapopulation dynamics (gene flow) or small population sizes in these naturally occurring early successional populations (factors that are likely not an issue in the restored populations in this study).

While the Lux population appears to have undergone rapid adaptation based on an integrated metric of survival and seed production, this metric does not include germination rate, which could affect fitness estimates and change the assessment of adaptation in these populations. I was unable to include germination data in aster models with the survival and seed data, as germination data came from a separate experiment in a different year. Rough estimates of *λ*, which include germination rate, were very low and showed few differences between populations, suggesting that despite the fact that germination rates did not significantly differ between populations (Figure 2a), germination can affect integrated fitness metrics. However, I interpret these *λ* estimates cautiously for two reasons. First, the germination rates in this study are surprisingly low compared to other field studies of *Chamaecrista* (e.g., Galloway & Fenster, 1999; Fenster & Galloway, 2000; Stanton‐Geddes, Shaw, & Tiffin, 2012; but see Sheth, Kulbaba, Pain, & Shaw, 2018), suggesting that there may have been an issue with the methods in the germination experiment or that germination rates in the particular year of the study are unlikely to represent long‐term patterns. Second, the very low growth rates suggest population decline, which may be the case for the Marshall population, but biomass estimates show no consistent decline in the Lux population (Figure S2). This suggests that these *λ* estimates may not accurately reflect population dynamics, at least for the Lux population, which we might expect given that it can be difficult to draw conclusions about population dynamics using demographic models parameterized with data collected over short time scales (Crone et al., 2011). Longer‐term demographic studies following both populations in this system, including all possible fitness components, would be needed to track population growth rates over time and determine not just whether rapid adaptation occurred in these populations, but whether it actually matters for population persistence.

If two closely located restored populations have different evolutionary outcomes, it suggests that the spatial scale of adaptation may inform restoration practice (McKay, Christian, Harrison, & Rice, 2005). Local seed sources are often assumed to be the most likely to be adapted to a restoration site (Johnson et al., 2010), given the prevalence of local adaptation in plants (Hereford, 2009; Leimu & Fischer, 2008; Oduor, Leimu, & Kleunen, 2016). However, recent studies have made the case for using multiple regional seed sources (Bucharova et al., 2019) or multiple local seed sources supplemented with nonlocal sources (Breed et al., 2018; Breed, Stead, Ottewell, Gardner, & Lowe, 2013), to maintain any regional adaptation while also increasing genetic diversity and therefore evolutionary potential. My study demonstrates that even closely located, similarly restored sites can vary enough to generate different evolutionary outcomes in the same source population. Give this fine‐scale environmental heterogeneity, using seed sources that increase adaptive potential may be the best approach to successfully establish populations in restorations. Increasing the genetic variation of restored populations in this way may also help populations adapt to changing climates (Breed et al., 2018; Harris, Hobbs, Higgs, & Aronson, 2006).

## 5 CONCLUSION

Although we often assume that adaptation will occur in populations colonizing novel habitats, this may not be the case, as adaptation can be constrained by a number of different variables that can be influenced by the nature of a colonization event. This study demonstrates that rapid adaptation may occur in ecological restorations, but that even geographically close populations restored under the same conditions can have different evolutionary outcomes. What remains to be seen is how commonly adaptation influences population establishment in restored habitats and other colonizing populations like range expansions and biological invasions. Although we are beginning to understand constraints to rapid adaptation and factors influencing evolutionary rescue in the laboratory (Bell, 2017), understanding what constrains rapid adaptation in nature is a key next step to overcoming these constraints to promote the establishment and persistence of restored populations.

## CONFLICT OF INTEREST

None declared.

## Supporting information

Supplementary Material

## Data Availability

Data from this study are archived in the Dryad Digital Repository, https://doi.org/10.5061/dryad.qnk98sfcj.

## References

[eva12959-bib-0001] Alexander, J. M. , Diez, J. M. , Hart, S. P. , & Levine, J. M. (2016). When climate reshuffles competitors: A call for experimental macroecology. Trends in Ecology and Evolution, 31, 831–841. 27640784 10.1016/j.tree.2016.08.003 PMC5159619

[eva12959-bib-0002] Antonovics, J. (1976). The nature of limits to natural selection. Annals of the Missouri Botanical Garden, 63, 224–247.

[eva12959-bib-0003] Bates, D. , Maechler, M. , Bolker, B. , & Walker, S. (2015). Fitting linear mixed‐effects models using lme4. Journal of Statistical Software, 67, 1–48.

[eva12959-bib-0005] Bell, G. (2017). Evolutionary rescue. Annual Review of Ecology, Evolution, and Systematics, 48, 605–627.

[eva12959-bib-0006] Bossdorf, O. , Auge, H. , Lafuma, L. , Rogers, W. E. , Siemann, E. , & Prati, D. (2005). Phenotypic and genetic differentiation between native and introduced plant populations. Oecologia, 144, 1–11. 15891837 10.1007/s00442-005-0070-z

[eva12959-bib-0007] Bradshaw, A. D. (1991). Genostasis and the limits to evolution. Philosophical Transactions of the Royal Society of London. Series B, Biological Sciences, 333, 289–305. 1682961 10.1098/rstb.1991.0079

[eva12959-bib-0008] Breed, M. F. , Harrison, P. A. , Bischoff, A. , Durruty, P. , Gellie, N. J. C. , Gonzales, E. K. , … Bucharova, A. (2018). Priority actions to improve provenance decision‐making. BioScience, 68, 510–516.

[eva12959-bib-0009] Breed, M. F. , Stead, M. G. , Ottewell, K. M. , Gardner, M. G. , & Lowe, A. J. (2013). Which provenance and where? Seed sourcing strategies for revegetation in a changing environment. Conservation Genetics, 14, 1–10.

[eva12959-bib-0010] Brooks, M. E. , Kristensen, K. , van Benthem, K. J. , Magnusson, A. , Berg, C. W. , Nielsen, A. , … Bolker, B. M. (2017). glmmTMB balances speed and flexibility among packages for zero‐inflated generalized linear mixed modeling. The R Journal, 9, 378–400.

[eva12959-bib-0011] Bucharova, A. , Bossdorf, O. , Hölzel, N. , Kollmann, J. , Prasse, R. , & Durka, W. (2019). Mix and match: Regional admixture provenancing strikes a balance among different seed‐sourcing strategies for ecological restoration. Conservation Genetics, 20, 7–17.

[eva12959-bib-0012] Colautti, R. I. , & Barrett, S. C. H. (2013). Rapid adaptation to climate facilitates range expansion of an invasive plant. Science, 342, 364–366. 24136968 10.1126/science.1242121

[eva12959-bib-0013] Colautti, R. I. , & Lau, J. A. (2015). Contemporary evolution during invasion: Evidence for differentiation, natural selection, and local adaptation. Molecular Ecology, 24, 1999–2017. 25891044 10.1111/mec.13162

[eva12959-bib-0014] Colautti, R. I. , Maron, J. L. , & Barrett, S. C. H. (2009). Common garden comparisons of native and introduced plant populations: Latitudinal clines can obscure evolutionary inferences. Evolutionary Applications, 2, 187–199. 25567860 10.1111/j.1752-4571.2008.00053.x PMC3352372

[eva12959-bib-0015] Connallon, T. , & Hall, M. D. (2018). Genetic constraints on adaptation: A theoretical primer for the genomics era. Annals of the New York Academy of Sciences, 1422, 65–87. 29363779 10.1111/nyas.13536

[eva12959-bib-0016] Crone, E. E. , Menges, E. S. , Ellis, M. M. , Bell, T. , Bierzychudek, P. , Erhlen, J. , … Williams, J. L. (2011). How do plant ecologists use matrix population models? Ecology Letters, 14, 1–8. 21070554 10.1111/j.1461-0248.2010.01540.x

[eva12959-bib-0017] Etterson, J. R. , & Shaw, R. G. (2001). Constraint to adaptive evolution in response to global warming. Science, 294, 151–154. 11588260 10.1126/science.1063656

[eva12959-bib-0018] Felker‐Quinn, E. , Schweitzer, J. A. , & Bailey, J. K. (2013). Meta‐analysis reveals evolution in invasive plant species but little support for Evolution of Increased Competitive Ability (EICA). Ecology and Evolution, 3, 739–751. 23531703 10.1002/ece3.488 PMC3605860

[eva12959-bib-0019] Fenster, C. B. (1991a). Gene flow in *Chamaecrista fasciculata* (Leguminosae) II. Gene establishment. Evolution, 45, 410–422. 28567871 10.1111/j.1558-5646.1991.tb04414.x

[eva12959-bib-0020] Fenster, C. B. (1991b). Gene flow in *Chamaecrista fasciculata* (Leguminosae) I. Gene dispersal. Evolution, 45, 398–409. 28567876 10.1111/j.1558-5646.1991.tb04413.x

[eva12959-bib-0021] Fenster, C. B. , & Galloway, L. F. (2000). Population differentiation in an annual legume: Genetic architecture. Evolution, 54, 1157–1172. 11005285 10.1111/j.0014-3820.2000.tb00551.x

[eva12959-bib-0022] Fox, J. , & Weisberg, S. (2011). An R companion to applied regression (2nd ed.). Thousand Oaks, CA: Sage.

[eva12959-bib-0023] Franks, S. J. , Avise, J. C. , Bradshaw, W. E. , Conner, J. K. , & Etterson, J. R. (2008). The resurrection initiative: Storing ancestral genotypes. BioScience, 58, 870–873.

[eva12959-bib-0024] Franks, S. J. , Sekor, M. R. , Davey, S. , & Weis, A. E. (2019). Artificial seed aging reveals the invisible fraction: Implications for evolution experiments using the resurrection approach. Evolutionary Ecology, 33(6), 811–824. 10.1007/s10682-019-10007-2

[eva12959-bib-0025] Gallagher, M. K. , & Wagenius, S. (2016). Seed source impacts germination and early establishment of dominant grasses in prairie restorations. Journal of Applied Ecology, 53, 251–263.

[eva12959-bib-0026] Galloway, L. F. , & Fenster, C. B. (1999). The effect of nuclear and cytoplasmic genes on fitness and local adaptation in an annual legume, *Chamaecrista fasciculata* . Evolution, 53, 1734–1743. 28565456 10.1111/j.1558-5646.1999.tb04558.x

[eva12959-bib-0027] Galloway, L. F. , & Fenster, C. B. (2000). Population differentiation in an annual legume: Local adaptation. Evolution, 54, 1173–1181. 11005286 10.1111/j.0014-3820.2000.tb00552.x

[eva12959-bib-0028] Geyer, C. J. (2018). aster: Aster models. R package version 1.0‐2. Retrieved from https://cran.r‐project.org/package=aster

[eva12959-bib-0029] Geyer, C. J. , Wagenius, S. , & Shaw, R. G. (2007). Aster models for life history analysis. Biometrika, 94, 415–426.

[eva12959-bib-0030] Grman, E. , Bassett, T. , Zirbel, C. R. , & Brudvig, L. A. (2015). Dispersal and establishment filters influence the assembly of restored prairie plant communities. Restoration Ecology, 23, 892–899.

[eva12959-bib-0031] Harris, J. A. , Hobbs, R. J. , Higgs, E. , & Aronson, J. (2006). Ecological restoration and global climate change. Restoration Ecology, 14, 170–176.

[eva12959-bib-0032] Hartig, F. (2019). DHARMa: Residual diagnostics for hierarchical (multi‐level/mixed) regression models. R package version 0.2.4. Retrieved from https://CRAN.R‐project.org/package=DHARMa

[eva12959-bib-0033] Hereford, J. (2009). A quantitative survey of local adaptation and fitness trade‐offs. The American Naturalist, 173, 579–588. 10.1086/597611 19272016

[eva12959-bib-0034] Huey, R. B. , Gilchrist, G. W. , Carlson, M. L. , Berrigan, D. , & Serra, L. (2000). Rapid evolution of a geographic cline in size in an introduced fly. Science, 287, 308–309. 10634786 10.1126/science.287.5451.308

[eva12959-bib-0035] Johnson, R. , Stritch, L. , Olwell, P. , Lambert, S. , Horning, M. E. , & Cronn, R. (2010). What are the best seed sources for ecosystem restoration on BLM and USFS lands? Native Plants, 11, 117–131.

[eva12959-bib-0036] Jump, A. S. , & Peñuelas, J. (2005). Running to stand still: Adaptation and the response of plants to rapid climate change. Ecology Letters, 8, 1010–1020. 10.1111/j.1461-0248.2005.00796.x 34517682

[eva12959-bib-0037] Kalisz, S. (1986). Variable selection on the timing of germination in *Collinsia verna* (Scrophulariaceae). Evolution, 40, 479–491. 28556331 10.1111/j.1558-5646.1986.tb00501.x

[eva12959-bib-0038] Keller, K. R. (2014). Mutualistic rhizobia reduce diversity and alter community composition. Oecologia, 176, 1101–1109. 25245262 10.1007/s00442-014-3089-1

[eva12959-bib-0039] Kinnison, M. T. , Unwin, M. J. , & Quinn, T. P. (2008). Eco‐evolutionary vs. habitat contributions to invasion in salmon: Experimental evaluation in the wild. Molecular Ecology, 17, 405–414. 17908221 10.1111/j.1365-294X.2007.03495.x

[eva12959-bib-0040] Kulpa, S. M. , & Leger, E. A. (2013). Strong natural selection during plant restoration favors an unexpected suite of plant traits. Evolutionary Applications, 6, 510–523. 23745142 10.1111/eva.12038 PMC3673478

[eva12959-bib-0041] LaRue, E. A. , Chambers, S. M. , & Emery, N. C. (2017). Eco‐evolutionary dynamics in restored communities and ecosystems. Restoration Ecology, 25, 19–26.

[eva12959-bib-0042] Lau, J. A. , Magnoli, S. M. , Zirbel, C. R. , & Brudvig, L. A. (2019). The limits to adaptation in restored ecosystems and how management can help overcome them. Annals of the Missouri Botanical Garden, 104, 441–454.

[eva12959-bib-0043] Leimu, R. , & Fischer, M. (2008). A meta‐analysis of local adaptation in plants. PLoS ONE, 3, e4010. 19104660 10.1371/journal.pone.0004010 PMC2602971

[eva12959-bib-0044] Leimu, R. , Mutikainen, P. , Koricheva, J. , & Fischer, M. (2006). How general are positive relationships between plant population size, fitness and genetic variation? Journal of Ecology, 94, 942–952.

[eva12959-bib-0045] Lenth, R. (2018). emmeans: Estimated marginal means, aka least‐squares means. R package version 1.3.1. Retrieved from https://CRAN.R‐project.org/package=emmeans

[eva12959-bib-0046] Macel, M. , Dostálek, T. , Esch, S. , Bucharová, A. , van Dam, N. M. , Tielbörger, K. , … Münzbergová, Z. (2017). Evolutionary responses to climate change in a range expanding plant. Oecologia, 184, 543–554. 28409227 10.1007/s00442-017-3864-x PMC5487849

[eva12959-bib-0047] Magnoli, S. M. , & Lau, J. A. (2019). Evolution in novel environments: do restored prairie populations experience strong selection? bioRxiv, 824409 10.1101/824409 32535882

[eva12959-bib-0048] Magnoli, S. M. , & Lau, J. A. (2020). Novel plant–microbe interactions: Rapid evolution of a legume–rhizobium mutualism in restored prairies. Journal of Ecology, 1–9. 10.1111/1365-2745.13366

[eva12959-bib-0049] McKay, J. , Christian, C. , Harrison, S. , & Rice, K. J. (2005). “How local is local?”—A review of practical and conceptual issues in the genetics of restoration. Restoration Ecology, 13, 432–440.

[eva12959-bib-0050] Mooney, H. A. , & Cleland, E. E. (2001). The evolutionary impact of invasive species. Proceedings of the National Academy of Sciences of the United States of America, 98, 5446–5451. 11344292 10.1073/pnas.091093398 PMC33232

[eva12959-bib-0051] Oduor, A. M. O. , Leimu, R. , & van Kleunen, M. (2016). Invasive plant species are locally adapted just as frequently and at least as strongly as native plant species. Journal of Ecology, 104, 957–968.

[eva12959-bib-0052] R Core Team . (2018). R: A language and environment for statistical computing. Vienna, Austria: R Foundation for Statistical Computing.

[eva12959-bib-0054] Rius, M. , & Darling, J. (2014). How important is intraspecific genetic admixture to the success of colonising populations? Trends in Ecology & Evolution, 29, 233–242. 24636862 10.1016/j.tree.2014.02.003

[eva12959-bib-0055] Sax, D. F. , Stachowicz, J. J. , Brown, J. H. , Bruno, J. F. , Dawson, M. N. , Gaines, S. D. , … Rice, W. R. (2007). Ecological and evolutionary insights from species invasions. Trends in Ecology & Evolution, 22, 465–471. 17640765 10.1016/j.tree.2007.06.009

[eva12959-bib-0056] Shaw, R. G. , & Etterson, J. R. (2012). Rapid climate change and the rate of adaptation: Insight from experimental quantitative genetics. New Phytologist, 195, 752–765. 22816320 10.1111/j.1469-8137.2012.04230.x

[eva12959-bib-0057] Shaw, R. G. , Geyer, C. J. , Wagenius, S. , Hangelbroek, H. H. , & Etterson, J. R. (2008). Unifying life‐history analyses for inference of fitness and population growth. The American Naturalist, 172, E35–47. 10.1086/588063 18500940

[eva12959-bib-0058] Sheth, S. N. , Kulbaba, M. W. , Pain, R. E. , & Shaw, R. G. (2018). Expression of additive genetic variance for fitness in a population of partridge pea in two field sites. Evolution, 72, 2537–2545. 30267420 10.1111/evo.13614

[eva12959-bib-0059] Stahlheber, K. A. , Watson, B. , Dickson, T. L. , Disney, R. , & Gross, K. L. (2016). Balancing biofuel production and biodiversity: Harvesting frequency effects on production and community composition in planted tallgrass prairie. Biomass and Bioenergy, 92, 98–105.

[eva12959-bib-0060] Stanton‐Geddes, J. , Shaw, R. G. , & Tiffin, P. (2012). Interactions between soil habitat and geographic range location affect plant fitness. PLoS ONE, 7, e36015. 22615745 10.1371/journal.pone.0036015 PMC3355151

[eva12959-bib-0061] Suding, K. N. (2011). Toward an era of restoration in ecology: Successes, failures, and opportunities ahead. Annual Review of Ecology, Evolution, and Systematics, 42, 465–487.

[eva12959-bib-0062] Urbanski, J. , Mogi, M. , O'Donnell, D. , DeCotiis, M. , Toma, T. , & Armbruster, P. (2012). Rapid adaptive evolution of photoperiodic response during invasion and range expansion across a climatic gradient. The American Naturalist, 179, 490–500. 10.1086/664709 22437178

[eva12959-bib-0063] van Kleunen, M. , Bossdorf, O. , & Dawson, W. (2018). The ecology and evolution of alien plants. Annual Review of Ecology, Evolution, and Systematics, 49, 25–47.

[eva12959-bib-0064] Vander Mijnsbrugge, K. , Bischoff, A. , & Smith, B. (2010). A question of origin: Where and how to collect seed for ecological restoration. Basic and Applied Ecology, 11, 300–311.

[eva12959-bib-0065] Walsh, B. , & Blows, M. W. (2009). Abundant genetic variation + strong selection = multivariate genetic constraints: A geometric view of adaptation. Annual Review of Ecology, Evolution, and Systematics, 40, 41–59.

[eva12959-bib-0066] Williams, J. L. , Hufbauer, R. A. , & Miller, T. E. X. (2019). How evolution modifies the variability of range expansion. Trends in Ecology & Evolution, 12, 903–913. 10.1016/j.tree.2019.05.012 31272695

[eva12959-bib-0067] Wise, M. J. , & Rausher, M. D. (2013). Evolution of resistance to a multiple‐herbivore community: Genetic correlations, diffuse coevolution, and constraints on the plant's response to selection. Evolution, 67, 1767–1779. 23730768 10.1111/evo.12061

[eva12959-bib-0068] Wood, C. W. , & Brodie, E. D. III (2015). Environmental effects on the structure of the G‐matrix. Evolution, 69, 2927–2940. 26462609 10.1111/evo.12795

