# Supplementary material for: Rapid adaptation (or not) in restored plant populations
Source: Evol Appl. 2020 Apr 23;13(8):2030–7. doi: 10.1111/eva.12959 (PMC7463322; doi:10.1111/eva.12959)

**Supplementary material**

**Table S1** Germination: ANOVA of the effects of plant population and site on seed germination. Bold type denotes a significant effect (p<0.05).

|  | χ^2^ | df | *P* |
| --- | --- | --- | --- |
| plant population | 2.15 | 2 | 0.34 |
| site | 0.12 | 1 | 0.72 |
| plant population*site | 0.94 | 2 | 0.63 |

**Table S2** Survival: ANOVA of the effects of plant population and site on plant survival. Bold type denotes a significant effect (p<0.05).

|  | χ^2^ | df | *P* |
| --- | --- | --- | --- |
| plant population | 2.54 | 2 | 0.28 |
| site | 87.15 | 1 | **<0.0001** |
| plant population*site | 4.62 | 2 | 0.10 |

**Table S3** Seed production: ANOVA of the effects of plant population and site on seed production (given survival). Bold type denotes a significant effect (p<0.05).

|  | χ^2^ | df | *P* |
| --- | --- | --- | --- |
| plant population | 8.23 | 2 | **0.02** |
| site | 35.44 | 1 | **<0.0001** |
| plant population*site | 4.22 | 2 | 0.12 |

**Figure S1** Photos of the (a) Lux and (b) Marshall sites. The Lux site is more grass-dominated and less productive than the Marshall site, which is more forb-dominated.


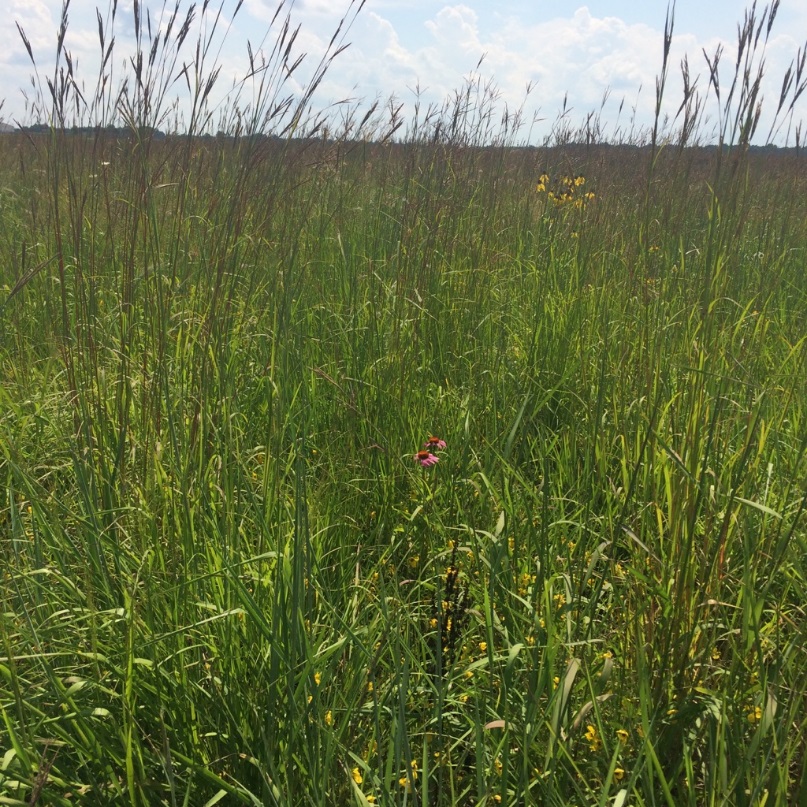
 (a)


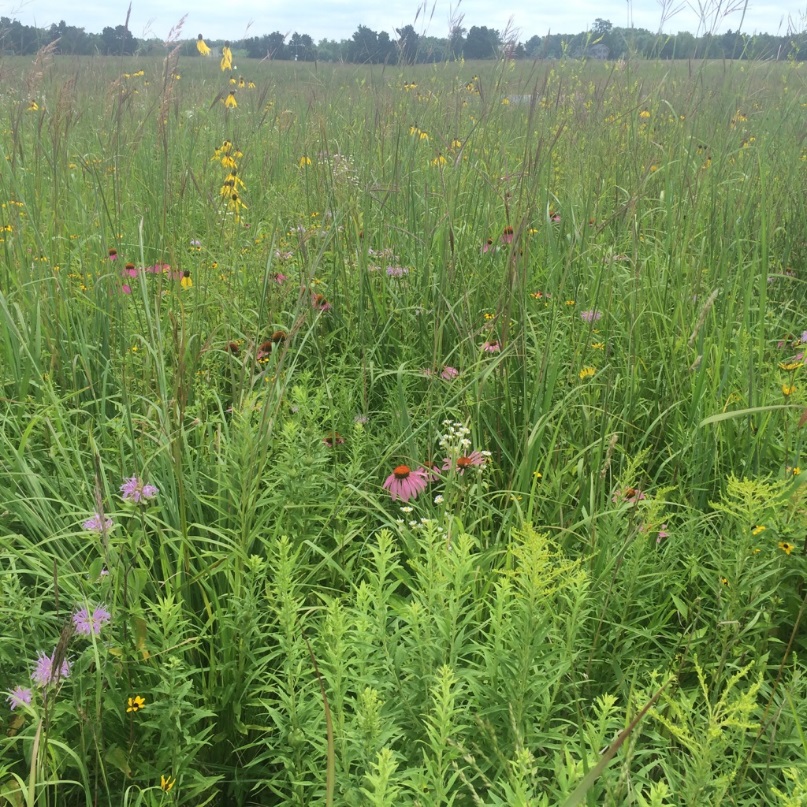
(b)

**Figure S2** Boxplots showing *Chamaecrista* biomass sampled from plots at each restoration site each year. All aboveground plant material was harvested from five 2 x 0.5 m quadrats at each site in August or September of each year. Plant material was sorted to the species level, dried at 60°C and weighed.


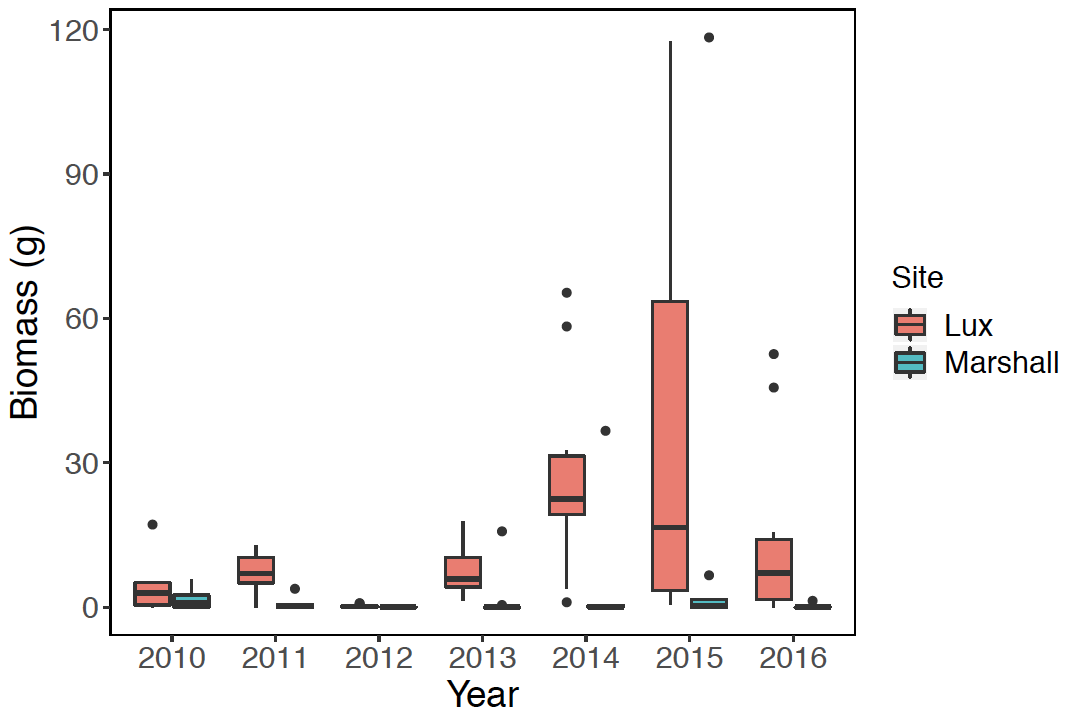

Supplement: Supplementary file 1 — Supplementary Material [file EVA-13-2030-s001.docx]
